# The association of dietary patterns with dietary inflammatory index, systemic inflammation, and insulin resistance, in apparently healthy individuals with obesity

**DOI:** 10.1038/s41598-021-86993-7

**Published:** 2021-04-06

**Authors:** Maryam Saghafi-Asl, Susan Mirmajidi, Mohammad Asghari Jafarabadi, Farhad Vahid, Nitin Shivappa, James R. Hébert, Vahideh Ebrahimzadeh Attari

**Affiliations:** 1 Department of Clinical Nutrition, School of Nutrition and Food Sciences, Tabriz University of Medical Sciences, Tabriz, Iran; 2 Student Research Committee, Tabriz University of Medical Sciences, Tabriz, Iran; 3 Department of Statistics and Epidemiology, School of Health, Tabriz University of Medical Sciences, Tabriz, Iran; 4 Road Traffic Injury Research Center, Tabriz University of Medical Sciences, Tabriz, Iran; 5 Luxembourg Institute of Health, Strassen, Luxembourg; 6 Department of Epidemiology and Biostatistics, Arnold School of Public Health, University of South Carolina, Columbia, SC 29208 USA; 7 Cancer Prevention and Control Program, Arnold School of Public Health, University of South Carolina, Columbia, SC 29208 USA; 8 Connecting Health Innovations, LLC, Columbia, SC 29201 USA; 9 Department of Nutrition and Food Sciences, Maragheh University of Medical Sciences, Margheh, Iran

**Keywords:** Medical research, Epidemiology

## Abstract

Inflammation is considered a key mechanism leading to obesity. Dietary patterns and certain food items influence inflammation. Few studies have investigated the contribution of major dietary patterns to biological measures of inflammation. Therefore, the present study aimed to examine the associations of different dietary patterns with dietary inflammatory index (DII), systemic inflammation, and insulin resistance (IR) in the apparently healthy obese. In this cross-sectional study, 151 abdominally obese subjects were recruited from the Northwest of Iran. Dietary intake, demographic data, anthropometric indices, and physical activity (PA) was assessed. DII scores were calculated based on a validated 168-item food frequency questionnaire (FFQ). Three dietary patterns were identified, using principal component analysis. Basal blood samples were collected to determine biochemical parameters. Linear regression test with adjusted beta estimates was applied for data analysis. Three dietary patterns were extracted as Healthy, Western, and Traditional. Body mass index (BMI) (p < 0.01) and fat mass (p < 0.001) were directly associated with the Western dietary pattern. Conversely, serum lipopolysaccharide-binding protein (LBP) (b = − 0.1, p < 0.04) was negatively associated with Healthy dietary pattern, after controlling for confounders. The Traditional pattern was found to be inversely related to DII (b = − 0.3, p < 0.001). The association was also reveresed between Traditional pattern and IR (Odds Ratio: 0.3 (95% Confidence Interval 0.1–0.9)). The results suggested that the Western dietary pattern was related to higher BMI and fat mass. In addition, the Healthy pattern was associated with decreased levels of LBP. Adherence to the Traditional dietary pattern was inversely related to DII as well as IR.

## Introduction

Obesity is a major public health concern, affecting more than half a billion people in the world^1^. It emerged as a threat in more wealthy countries nearly four decades ago^2^. Globally, the pandemic of overweight and obesity in adults (i.e., those with body mass index (BMI) of ≥ 25 kg/m^2^) has increased from 28.8 to 36.9% in men and from 29.8 to 38.0% in women during the years 1980–2013^3^. Over half of the Iranian adult population are overweight or obese. The prevalence of obesity and abdominal obesity increased from 23.1% and 47.9% in 1999 to 34.1% and 71.1% in 2011, respectively^4,5^.

Risk factors associated with obesity are multiple and complex. The most closely related adverse health consequences include type 2 diabetes mellitus (T2DM), dyslipidemia, cardiovascular diseases (CVD), and insulin resistance (IR)^6^. There is a strong direct association between obesity and IR in nondiabetic subjects. Obesity also increases IR in diabetic subjects and may lead to enhanced acute-phase response^7^. Overweight and obesity are characterized by chronic low-grade inflammation that results in the secretion of pro-inflammatory factors engaged in the pathogenesis of IR^8^. In addition, an abundance of adipose mass can result in the secretion of inflammatory adipokines^9^.

Chemerin is a novel inflammatory adipokine that has a regulatory role in lipid and glucose homeostasis^10^. Moreover, there is a positive correlation between plasma concentration of chemerin and body mass index (BMI), triglycerides, and blood pressure^11^. A body of evidence shows that chemerin concentration is increased in overweight and obese persons. These conditions exhibit a positive correlation with various aspects of metabolic syndrome.

Omentin, a 38–40 kDa adipokine, was observed from a cDNA in visceral omental adipose tissue^12^. Serum omentin levels may play a key role in the pathogenesis of IR and diabetes^13^. Omentin can increase insulin sensitivity and stimulate glucose transport and Akt phosphorylation in human adipocytes^14^. Furthermore, some studies have demonstrated that circulating omentin was most notably decreased in obesity^15^.

Lipopolysaccharide binding protein (LBP), as an acute-phase protein, is a 50 kDa polypeptide that is commonly synthesized in the liver^16^. Serum LBP level is a marker of coronary artery disease^17^. It is reported to be associated with obesity and related disorders in apparently healthy people^18^. Lipopolysaccharide stimulates the release of several cytokines that are key inducers of IR which is a putative factor for triggering metabolic disturbances^19^. A recent study indicated that increased metabolic concentrations of plasma LPS are sufficient molecular mechanisms for the development of IR, obesity, and T2DM^20^.

Diet is an important predictor of circulating levels of inflammatory markers^21^. Diets rich in pro-inflammatory constituents such as saturated fatty acids (SFAs) and trans fatty acids have consistently been associated with proliferation^22^ and oxidative stress that can promote inflammation^23^. By contrast, polyunsaturated fatty acids (PUFAs), monounsaturated fatty acids (MUFAs), and fiber have been shown to attenuate the inflammatory cascade^24^. Recently, a survey on the inflammatory potential of diet and its influence on obesity and chronic diseases has received special attention^25^.

The dietary inflammatory index (DII) is a novel scoring algorithm that provides an estimate of the inflammatory potential of the overall diet based on the inflammatory properties of dietary constituents^26^. Food pattern analysis is a way to investigate the relationship between diet and risk of chronic diseases^27^. Currently, few studies have studied the association between dietary patterns and DII. Dietary patterns with more protein, specifically animal protein may also aggravate glucose metabolism, leading to the development of IR^28^. Moreover, a body of evidence shows that certain dietary patterns have also been associated with the markers of inflammation^29^. A cross-sectional study of the Hispanic elderly living in Massachusetts reported lower concentrations of CRP with higher fruit and vegetable consumption^30^. To our knowledge, there exists no study that evaluates the association of different dietary patterns with DII, systemic inflammation, and IR in apparently healthy obese individuals in the Middle East. Therefore, the present study was aimed to identify the dietary patterns of apparently healthy obese and to determine their association with DII as well as measures of systemic inflammation and IR in Iran.

## Materials and methods

### Sampling

The present cross-sectional study was conducted from June to November 2015 in Tabriz, Iran. Five-hundred volunteers were randomly invited from the general population. Of these, 151 apparently healthy obese (BMI ≥ 30 kg/m^2^) people (aged 18–60 years) were consecutively enrolled, based on defined eligibility criteria^31,32^. Exclusion criteria included having any chronic disease such as hypertension, diabetes, CVD, hepatic disorders, renal diseaseor gastrointestinal disease; surgery during the past year; any current cancer, infectious disease, or severe mental illness. Moreover, those on a diet or who misreported dietary intake (< 800 kcal/day or > 4200 kcal/day) or were taking any medications that could influence lipid and glucose metabolism, corticosteroids, contraceptives, antibiotics, anti-obesity drugs, anti-inflammatory drugs, beta-blockers, anti-coagulants or any diettary supplements including multivitamins, multiminerals, antioxidants, herbal supplements, and omega 3 or omega 6 fatty acids in the last two months were also excluded. The study was approved by the ethics committee of Tabriz University of Medical Sciences, Tabriz, Iran. Informed consent was obtained from all the atudy individuals. The whole research was performed in compliance with the Declaration of Helsinki (Ethical code: TBZMED.REC.1395.1169).

### Measurements

Height, weight, WC, and hip circumference (HC) were measured, while the participant wore clothes without shoes. Body mass index (BMI) was estimated as weight in kilograms divided by the square of height in meters. WC was measured at the midpoint between the lower ribs and the iliac crest and HC was measured at the level of the maximum extension of the buttocks. WHR was calculated as WC divided by HC^33^. Physical activity (PA) data was extracted from long-form International Physical Activity Questionnaire (IPAQ)^34^. PA was expressed as metabolic equivalents (Mets)-h per week.

### Laboratory assays

Blood samples were drawn after an overnight fast at the beginning of the study. The serum was centrifuged at 3000 rpm for 15 min. Lipid profile and blood sugar were calculated immediately, using the commercial kits (Pars Azmoon, Tehran, Iran) and a Selectra 2 auto-analyzer (Vital Scientific, Spankeren, the Netherlands). Inter-assay and intra-assay coefficients of variation (CVs) were < 5% for all assays. Serum samples were stored at − 80 °C, until analysis. Sandwich Enzyme-Linked Immunosorbent Assay (9) was used to measure serum insulin, LBP, chemerin, and omentin concentrations (Monobind Inc., Lake Forest, CA, USA). Other parameters were measured according to the manufacturer’s instructions (Bioassay Technology Laboratory, Shanghai Korean Biotech, Shanghai City, China). The intra-assay and inter-assay CVs were < 8% and 10% for chemerin, omentin, and LBP and < 8% and 9.8% for insulin, respectively. The homeostasis model assessment of IR (HOMA-IR) was calculated, using fasting plasma glucose and fasting insulin values, according to the formula.

### Assessment of dietary intake

The food intake of the indivuals during last year was assessed, using a validated semi-quantitative food frequency questionnaire (FFQ)^34^. The FFQ consisted of 168 food items and the participants reported their frequency of intake of each food item on a daily, weekly, monthly, or yearly basis^35^. Then, daily grams of food intake were estimated from food intake data. The *Nutritionist IV* software (Axxya Systems, Stafford, TX, 1994), modified for Iranian foods was used to calculate the energy and nutrient contents of foods^36^. Foods from FFQ were categorised into 29 food groups, on the basis of nutrient profile or culinary usage (Appendix 1).

Food patterns, consisting of 29 food groups were analyzed. The principal component analysis was used to identify explanatory factors^37^. Three dietary patterns were recognized, according to eigen value (> 1), scree plot, factor interpretability, and the variance explained (> 5%). Factors were rotated with varimax rotation to develop the interpretability of the factors and minimize the correlation between them. Factor loadings are equivalent to the correlation between food items and factors. Higher loadings represent a higher shared variance with the factor. Factor loadings of > 0.20 indicated the foods that were significantly related to the identified factor^37,38^. After recognizing dietary patterns, the participants were assigned, based on their patterns factor score. Factor scores were divided into quartiles in terms of their distribution in each stratum, implying increased intake from tertile 1 to tertile 3.

The DII scores were calculated according to FFQ questionnaire^26^. Briefly, dietary data for each participant were first linked to a regionally representative global database that provided a robust estimate of means and standard deviations for each of the food parameters considered (i.e. foods, nutrients, and other food components).The ‘standard mean’ was subtracted from the actual food parameter value and divided by its standard deviation. This z-score was then converted to a percentile (so as to minimize the effect of outliers or right-skewing, a common occurrence with dietary data). This value was then converted to a proportion (values 0–1) and centered by multiplying by 2 and subtracting one. This value was then multiplied by the respective inflammatory effect score of the food parameters (derived from a literature review and scoring of 1943 ‘qualified’ articles) to obtain the subject’s food parameter-specific DII score. All of the food parameter-specific DII scores were then summed to create the overall DII score for each subject in the study.

### Sample size estimation

Five persons per group are recommended for factor analysis^39^; hence, 145 people were adequate to be recruited based on 29 food groups in the present study. However, 150 apparently healthy obese were recruited for this purpose.

### Statistical analysis

Statistical Package for the Social Sciences (SPSS) software (Version 25, SPSS, Inc., Chicago, IL, USA) was applied to perform data analysis. Twenty-one cases of study participants were excluded because of the missing data; the final analysis included 150 participants. The Kolmogorov- Smirnov test was used to check the normality of data distribution. The characteristics of the participants are expressed as means (standard deviation) for continuous variables and percentages for categorical variables. We used multiple linear regression analysis to find out the association of dietary patterns with systemic inflammatory markers and DII. All models were adjusted for sex, weight, and energy intake (continuous). In these models, scores of the dietary pattern were the independent variable (grouped as tertile of the dietary pattern) and HOMA-IR was dependent variable (IR^+^ vs. IR^−^). These models were adjusted for variables found to be significantly associated with IR such as age, sex, energy intake, physical activity, and level of education.

### Ethical approval

Dr. James R. Hébert owns a controlling interest in Connecting Health Innovations LLC (CHI), a company that has licensed the right to his invention of the dietary inflammatory index (DII) from the University of South Carolina in order to develop computer and smartphone applications for patient counseling and dietary intervention in clinical settings. The subject matter of this paper will not have any direct bearing on that work, nor has that activity exerted any influence on this project. Dr. Nitin Shivappa is an employee of CHI.

## Results

Three major patterns were identified, using factor analysis (Appendix 2): the Healthy dietary pattern (higher intakes of yellow vegetables including carrots, as row orboiled, nuts, unsweetened drinks, tomato, grains (including White breads (lavash, baguette), whole breads (sangak, barbari, taftoon), noodles, pasta, rice, biscuit, barley, bulgur, cornflakes, olive, low-fat dairy, eggs, fish, legumes, potato, and dry fruits); the Western dietary pattern (higher intakes of mayonnaise, high-fat dairy, soft drinks, butters, fruit juices, French fries, pizza, sweets, processed meat, and pickles); and the Traditional dietary pattern (higher intakes of poultry, organ meats, fruits (whole, juice, or puree), green vegetables (including green leafy vegetables such as lettuce, spinach, as row or boiled), other vegetables (including Cabbage, cauliflower, Brussels sprouts, kale, cucumbers, mixed vegetable, eggplant, celery, green peas, green beans, green peppers, corn, turnips, squash, mushrooms, onions, and garlic), red meats, and hydrogenated fat. Together, these factors explained 30% of the variance.

Characteristics of the study participants across tertile categories of food patterns scores are presented in Table 1. Compared with participants in the lower tertile, those in the upper tertile of the Healthy and Traditional dietary patterns had lower energy intake. Unlike females, male patients presented higher adherence to the upper tertile of the Healthy pattern. Those in the upper tertile of the Western dietary pattern had higher BMI, fat mass, energy intake, and lower carbohydrate intake and had a lower age.

**Table 1:** Descriptive characteristics of the study population and their association with tertile of dietary patterns.

	Healthy			${2}_{\mathrm{p}}$	Traditional			${2}_{\mathrm{p}}$	Western			${2}_{\mathrm{p}}$
	< 47.46	47.47–72.93	> 72.93		< 9.87	9.88–14.64	> 14.65		< 5.20	5.21–12.02	> 12.03	
Age (years)	37 ± 8.5	36 ± 8.0	34 ± 8.7	0.20	37 ± 7.5	38 ± 9.1	37 ± 8.8	0.79	39 ± 8.5	37 ± 8.5	34 ± 7.7	0.01
**Sex**				< 0.001				0.22				0.53
Male	16 (32%)	24 (49%)	34 (69.4%)		24 (49%)	29 (59.2%)	74 (72%)		22 (44.9%)	28 (56%)	24 (49%)	
Female	34 (68%)	25 (51%)	15 (30.6%)		25 (51%)	20 (40.8%)	29 (28%)		27 (55.1%)	22 (44%)	25 (51%)	
BMI (kg/m^2^)	31.3 ± 3.7	32.0 ± 5.0	31.8 ± 3.7	0.68	32.0 ± 4.5	31.5 ± 3.6	31.6 ± 4.4	0.86	31.9 ± 4.1	30.4 ± 3.8	32.9 ± 4.3	0.01
Fat Mass	28.3 ± 7.9	28.6 ± 10.3	27.2 ± 7.2	0.68	28.0 ± 9.1	27.5 ± 8.1	28.6 ± 8.4	0.81	28.3 ± 8.3	25.4 ± 8.3	30.5 ± 8.4	0.001
**PA (MET-h/d)**				0.29				0.22				0.10
< 600	2 (4.2%)	8 (18.2%)	6 (12.5%)		2 (4.3%)	8 (17.8%)	6 (12.2%)		4 ( (8.9%)	2 (4.3%)	10 (20.4%)	
600–3000	22 (45.8%)	19 (43.2%)	19 (39.6%)		18 (39.1%)	19 (42.2%)	23 (46.9%)		18 (40%)	24 (52.2%)	18 (36.7%)	
> 3000	24 (50%)	17 (38.6%)	23 (47.9%)		26 (56.5%)	18 (40.0%)	20 (40.8%)		23 (51.1%)	20 (43.5%)	21 (42.9%)	
**Education**				0.99				0.46				0.40
Middle school	13 (26%)	12 (24.5%)	12 (24.5%)		13 (26.5%)	15 (30.6%)	9 (18%)		9 (18.1%)	16 (32%)	12 (24.5%)	
High school	23 (46%)	23 (46.9%)	23 (46.9%)		20 (40.8%)	21 (42.9%)	28 (56%)		26 (53.1%)	23 (46%)	120 (40.8%)	
University & Higher education	14 (28%)	14 (28.6%)	14 (28.6%)		16 (32.7%)	13 (26.5%)	13 (26%)		14 (28.6%)	11 (22%)	17 (24.7%)	
Fat% (%E)	27.9 ± 9.2	27.0 ± 10.9	24.8 ± 8.1	0.28	25.9 ± 8.0	26.2 ± 10.6	227.3 ± 9.5	0.79	25.0 ± 10.54	26.2 ± 7.7	28.5 ± 9.5	0.22
CHO% (%E)	59.0 ± 9.1	59.1 ± 10.3	62.2 ± 9.2	0.45	61.1 ± 8.4	61.1 ± 10.5	59.2 ± 9.3	0.75	63.3 ± 9.2	59.1 ± 8.1	57.2 ± 9.4	0.02
Pro% (%E)	14.2 ± 3.3	13.7 ± 2.8	14.8 ± 3.9	0.40	14.6 ± 3.2	14.3 ± 3.8	13.9 ± 3.2	0.67	13.4 ± 3.1	14.5 ± 2.9	15 ± 4.1	0.09
Energy intake (kcal/day)	2399 ± 803	3030 ± 966	3562 ± 677	0.001	2389 ± 734	2999 ± 915	3591 ± 79	0.001	2453 ± 775	3107 ± 939	3410 ± 876	0.001

*%E* percent of energy.

Data are presented as means Sd for continuous variables and n (%) foe categorical variables.

^a^ANOVA for quantitative variables.

^b^Chi-square for qualitative variables.

The results of multiple linear regression models are shown in Table 2. The Healthy pattern score was inversely related to plasma concentration of LBP, after controlling for potential confounders. The Traditional pattern score was negatively associated with DII when controlled for confounders including sex, weight, and energy intake.

**Table 2:** Linear regression analysis for the association between dietary patterns and circulating concentrations of inflammatory markers and DII.

Inflammatory markers and DII score	Healthy		Traditional		Western	
	β	p	β	p	β	p
Omentin (ng/mL)	0.10	0.28	− 0.03	0.78	0.05	0.54
Chemerin (ng/mL)	0.001	0.84	− 0.001	0.99	− 0.15	0.09
Insulin (mg/dL)	0.18	0.48	− 0.13	0.19	− 0.11	0.20
LBP (µg/mL)	− 0.18	0.04	0.05	0.62	0.12	0.16
DII score	− 0.13	0.15	− 0.37	0.001	− 0.11	0.19

The model was adjusted for energy intake, weight, and sex.

*LBP* lipopolysaccharide binding protein, *DII* dietary inflammatory index.

The results of logistic regression analysis indicated that the highest tertile of the Traditional dietary pattern was associated with approximately 70% decreased odds of IR, as indicated by HOMA-IR (OR: 0.32, 95% CI 0.11–0.93) (Table 3). The Western or Healthy dietary pattern showed no significant association with the odds of IR in the study population.

**Table 3:** Adjusted logistic regression model describing the association between various dietary patterns and insulin resistance in the study population.

Tertiles of dietary patterns	Unadjusted model OR; 95% CI	Adjusted model OR; 95% CI
**Healthy**		
1	Ref	Ref
2	1.5 (0.6–3.4)	2.1 (0.8–5.3)
3	1.5 (0.7–3.5)	2.3 (0.8–6.5)
**Traditional**		
1	Ref	Ref
2	1 (0.4–2.3)	0.6 (0.2–1.7)
3	0.7 (0.3–1.6)	0.3 (0.1–0.9)
**Western**		
1	Ref	Ref
2	0.9 (0.4–2)	0.9 (0.3–2.3)
3	0.9 (0.4–2.1)	0.5 (0.1–1.3)

Adjusted for age, sex, energy intake, physical activity, and education.

## Discussion

In the present study, three major dietary patterns were identified, as follows: Healthy, Western, and Traditional. Of these, the Healthy pattern was inversely associated with serum LBP concentration. Also, a positive association between the Western diet and BMI as well as fat mass was found. The Traditional pattern was inversely associated with IR and DII.

The present work showed that LBP was the only biomarker to appear inversely associated with a Healthy dietary pattern; i.e. serum LBP was lower in those with Healthy dietary pattern. The finding for LBP is consistent with the notion that Healthy diets afford protective effects from diverse perspectives. One plausible mechanism is through a TLR4-dependent pathway, implicated in the launch of the transcription factor, nuclear factor-қB (NFқB)^40^. This signaling cascade results in the enhanced release of pro-inflammatory cytokines, including tumor necrosis factor-alpha (TNF-α) or IL-6, thus promoting systemic inflammation^41^. Endotoxin levels have indicated a direct correlation with IR and chemerin and an inverse association with omentin^42^. The negative correlation between serum LBP and chemerin was also observed in our previous study^25^.

Other studies reported a link between a high-fat diet especially saturated fatty acids and the release of endotoxins in the plasma of mice and humans^21–43^. In this regard, López-Moreno et al.^43^ showed that consumption of diets with highly-saturated fatty acids (HSFAs) increases the intestinal absorption of LPS which, in turn, increases postprandial endotoxemia levels and the postprandial inflammatory response. In fact, chronic consumption of a high-fat diet especially rich in SFAs as well as obesity could increase endotoxemia and low-grade inflammation due to the repeated endotoxin absorption from the gut through the digestion of lipids which, in turn, could raise the risk of IR and atherosclerosis^44^. It was also reported that fruits intake and a fiber-rich meal reduce postprandial LPS levels, as compared with a high-fat, especially saturated, meal in human. Moreover, it was shown that the n-3 fatty acid-rich test meal reduces postprandial serum endotoxin, compared to the meal rich in saturated fatty acid^43^. However, in the present study, those with the Healthy dietary pattern had a lower intake of fat (i.e. saturated and trans fatty acids as well as dairy products) and subsequently, lower levels of serum LBP. No other correlation was found between the Healthy dietary pattern and other biomarkers.

Our findings also confirm a contrary association between the Traditional pattern and DII. It was also identified that those in the highest tertile of the Traditional diet had approximately 70% lower HOMA-IR scores, compared to the individuals in the lower tertile. The Traditional diet mainly included poultry, fruit, green leafy vegetables, red meat, organ meat, and hydrogenated fat which taken together could be responsible for the lower IR and DII in the study population. Higher consumption of fruit and vegetables in the Traditional diet could be considered as the key factor causing the inverse association between the Traditional dietary pattern, DII, and IR. The main ingredients of fruit and vegetables are dietary fiber, vitamin E, folate, and magnesium which are independently associated with reduced metabolic risks^44^. Reduced insulin demand may be another protective mechanism associated with higher intakes of these foods. In general, due to physical properties and viscous structures of fibers, such foods are primarily non-digestible carbohydrates and absorbed more slowly; thus, they have relatively low glycemic indices.

Regarding the consumption of meat, a cross-sectional analysis of data from 3690 diabetes-free female participants in the Nurses’ Health study found that a greater total, unprocessed, and processed red meat intakes were associated with higher inflammatiry biomarkers. Substitution of a serving of total red meat intake with alternative protein food consumed in a combination of poultry, fish, legumes, and nuts was associated with a healthier biomarkers profile of inflammation^45^. However, a further cross-sectional study demonstrated that the relationship between red meat intake and inflammatory biomarkers was no longer observed after adjustment for BMI^46^. It was found that a pro-inflammatory diet, defined as a higher DII score, is cheifly rich in some inflammatory ingredients such as SFA, and comparatively, poor in anti-inflammatory parameters including fibers, MUFA, and PUFA^25^.

Our previous work indicated that participants with higher DII score had lower intake of polyunsaturated and monounsaturated fats and fiber as well as higher saturated fats^25^. Similarly, Shivappa et al*.* reported a negative association between lower DII score and consumption of healthy foods and nutrients^47^. Shivappa et al*.*^47^ also illustrated that a diet rich in pro-inflammatory food parameters such as saturated fatty acids and moderately poor in anti-inflammatory food ingredients such as fruit and vegetables can increase inflammatory biomarkers, as evidenced by the increased levels of IL-6 and homocysteine. In line with our research, another study suggested that a food pattern rich in fiber was indirectly associated with hyperinsulinemia in women, but not in men^48^. In the Health Professionals Follow-up Study, a prudent dietary pattern full of fruits, vegetables, whole grains, and poultry was inversely associated with insulin levels^49^, which is consistent with our results. In a UK cross-sectional study, the Isle of Ely Study, a dietary pattern poor in fruits and vegetables and rich in processed meats and French fries was associated with previously diagnosed diabetes^50^.

A direct association between the Western diet and BMI (including fat mass) is in line with previous researches. Both cross-sectional^51^ and prospective studies^52^ have reported the parallel findings. A ‘‘meats’’ dietary pattern including “meat”, rich in processed and red meats, fish, poultry, eggs, fats and oils, and condiments was correlated with higher BMI in a group of Hawaiian women^51^. A direct association between Western dietary patterns and obesity was also confirmed by Slattery et al*.*^53^*.* In an 8-year prospective study among 50,000 adult women, Schulz et al*.*^54^ demonstrated that Western dietary pattern was attributed to increased weight gain and higher intakes of red and processed meats and sweets. Our results are similar to this study showing an association of Western patterns with higher BMI and fat mass. It is noteworthy that their Western pattern was reflected in diets high in red and processed meats, refined grains, sweets and desserts, and potatoes which was like to ours. Joung et al*.*^55^ reported that diets containing processed and red meats, oils or fats including (corn oil, margarin, butter, sesame oil, and soybean oil), and sugar may be correlated with obesity in Korean adults.

Levels of plasma leptin reflect the amount of fat stored in adipose tissue. Leptin has a key role in energy homeostasis. Circulating leptin increases by 40% after acute overfeeding, whereas fasting is correlated with significantly decreased leptin secretion^56^. Also, Western dietary pattern principally characterized by highly saturated fat was found to be directly associated with higher leptin concentration^56^. It is noteworthy to mention that our Western pattern was similarto those of Western or unhealthy dietary patterns in different studies^49–58^.

The present study contains several strengths. It used a validated FFQ. Moreover, to our knowledge, this is the first to identify the association of dietary patterns of the obese with DII and inflammatory parameters. The DII has previously been validated in the Iranian population^59^. This tool is standardized for food intake from various populations around the world^25–60^; therefore, scores in Iran can be compared with those obtained in other populations. It should be noted, however, that the DII was calculeted with data from 32 of the 45 food variables, thus 13 parameters are missing in our study. In fact, a total of 32 variables (including energy, carbohydrate, protein, total fat, fiber, cholesterol, saturated fat, mono-unsaturated fat, poly unsaturated fat, omega-3, omega-6, niacin, thiamin, riboflavin, vitamin B12, vitamin B6, iron, magnesium, selenium, zinc, vitamin A, vitamin C, vitamin D, vitamin E, folic acid, beta carotene, garlic, ginger, onion, turmeric, saffron, pepper) were available from the 45-item FFQ which could be used to compute DII. An additional limitation should be noted; because of the cross-sectional nature of our study,it is not possible to infer anything about the temporal component of a putative cause-effect relationship. In addition, measurement accuracy errors inherent in the use of FFQs for dietary assessment may exist^61,62^.

## Conclusion

The results suggested that Western dietary patterns, including higher intakes of red meat and sweets, was related to greater BMI and fat mass. In addition, the Healthy pattern with lower intake of SFA and trans fatty acids led to a significant reduction in the proinflammatory markers i.e. LBP. Adherence to Traditional dietary pattern rich in fruit and vegetables was inversely related to DII as well as IR. Altogether, it seems that dietary patterns can affect systemic inflammation, either directly or indirectly. Further studies with larger sample size are warranted to investigate the associations of diet-induced inflammation with altered levels of adipokines as well as IR markers.

## Supplementary Information

Supplementary Information.

## Abbreviations

BMI: Body mass index
CVD: Cardiovascular disease
DII: Dietary inflammatory index
ELISA: Enzyme-Linked Immunosorbent Assay
FFQ: Food frequency questionnaire
HC: Hip circumference
HDL-C: High-density lipoprotein-cholesterol
HOMA-IR: Homeostasis model assessment of insulin resistance
IL18: Interleukin 18
IPAQ: International Physical Activity Questionnaire
IR: Insulin resistance
LBP: Lipopolysaccharide-binding protein
LPS: Lipopolysaccharide
MUFA: Monounsaturated fatty acid
MetS: Metabolic Syndrome
NFқB: Nuclear factor-қB
PUFA: Polyunsaturated fatty acid
PA: Physical activity
SFA: Saturated fatty acid
TLR: Toll-like receptor
TNF-α: Tumor necrosis factor-alpha
WC: Waist Circumference

## References

[1] Bhurosy T, Jeewon R (2014). Overweight and obesity epidemic in developing countries: A problem with diet, physical activity, or socioeconomic status?. Sci. World J..

[2] Fleming T, Robinson M, Thomson B, Graetz N, Margono C (2014). Global, regional, and national prevalence of overweight and obesity in children and adults during 1980–2013: A systematic analysis for the Global Burden of Disease Study 2013. Lancet.

[3] Vandevijvere S, Chow CC, Hall KD, Umali E, Swinburn BA (2015). Increased food energy supply as a major driver of the obesity epidemic: A global analysis. Bull. World Health Organ..

[4] Rashidi A, Mohammadpour-Ahranjani B, Vafa M, Karandish M (2005). Prevalence of obesity in Iran. Obes. Rev..

[5] Barzin M (2015). Rising trends of obesity and abdominal obesity in 10 years of follow-up among Tehranian adults: Tehran Lipid and Glucose Study (TLGS). Public Health Nutr..

[6] Reaven GM (1998). Role of insulin resistance in human disease. Diabetes.

[7] Kahn B, Flier JS (2000). Obesity and insulin resistance. J. Clin. Invest..

[8] Bastard J-P (2006). Recent advances in the relationship between obesity, Inflammation, and insulin resistance. Eur. Cytokine. Netw..

[9] Chang SS, Eisenberg D, Zhao L, Adams C, Leib R, Morser J (2016). Chemerin activation in human obesity. Obesity.

[10] Haberl EM (2018). Ex vivo analysis of serum chemerin activity in murine models of obesity. Cytokine.

[11] Bozaoglu K (2007). Chemerin is a novel adipokine associated with obesity and metabolic syndrome. Endocrinology.

[12] Yamawaki H, Tsubaki N, Mukohda M, Okada M, Hara Y (2010). Omentin, a novel adipokine, induces vasodilation in rat isolated blood vessels. Biochem. Biophys. Res. Commun..

[13] Fu M (2004). Systematic analysis of omentin 1 and omentin 2 on 1q23 as candidate genes for type 2 diabetes in the Old Order Amish. Diabetes.

[14] Yang R-Z (2006). Identification of omentin as a novel depot-specific adipokine in human adipose tissue: Possible role in modulating insulin action. Am. J. Physiol. Endocrinol. Metab..

[15] de Souza Batista CM (2007). Omentin plasma levels and gene expression are decreased in obesity. Diabetes.

[16] Zweigner J, Schumann RR, Weber JR (2006). The role of lipopolysaccharide-binding protein in modulating the innate immune response. Microbes. Infect..

[17] Gubern C (2006). Natural antibiotics and insulin sensitivity: The role of bactericidal/permeability-increasing protein. Diabetes.

[18] Sun L (2010). A marker of endotoxemia is associated with obesity and related metabolic disorders in apparently healthy Chinese. Diabetes Care.

[19] Moreno-Navarrete J (2012). Circulating lipopolysaccharide-binding protein (LBP) as a marker of obesity-related insulin resistance. IJO..

[20] Cani PD (2007). Metabolic endotoxemia initiates obesity and insulin resistance. Diabetes.

[21] Ahluwalia N, Andreeva V, Kesse-Guyot E, Hercberg S (2013). Dietary patterns, inflammation and the metabolic syndrome. Diabetes. Metab..

[22] Vykhovanets EV, Shankar E, Vykhovanets OV, Shukla S, Gupta S (2011). High-fat diet increases NF-jB signaling in the prostate of reporter mice. Prostate.

[23] Ma Y (2006). Association between dietary fiber and serum C-reactive protein. Am. J. Clin. Nutr..

[24] van Woudenbergh GJ (2013). Adapted dietary inflammatory index and its association with a summary score for low-grade inflammation and markers of glucose metabolism: the Cohort study on Diabetes and Atherosclerosis Maastricht (CODAM) and the Hoorn study. Am. J. Clin. Nutr..

[25] Mirmajidi S (2018). Inflammatory potential of diet: Association with chemerin, omentin, lipopolysaccharide-binding protein, and insulin resistance in the apparently healthy obese. J. Am. Coll. Nutr..

[26] Shivappa N, Steck SE, Hurley TG, Hussey JR, Hébert JR (2014). Designing and developing a literature-derived, population-based dietary inflammatory index. Public Health Nutr..

[27] Martinez-Gonzalez MA (2009). Mediterranean food pattern and the primary prevention of chronic disease: Recent developments. Nutr. Rev..

[28] Duc S (2005). Anthropometric characteristics, dietary patterns and risk of type 2 diabetes mellitus in Vietnam. J. Am. Coll. Nutr..

[29] Lopez-Garcia E (2004). Major dietary patterns are related to plasma concentrations of markers of inflammation and endothelial dysfunction. Am. J. Clin. Nutr..

[30] Gao X, Bermudez OI, Tucker KL (2004). Plasma C-reactive protein and homocysteine concentrations are related to frequent fruit and vegetable intake in Hispanic and non-Hispanic white elders. J. Nutr..

[31] Meigs JB (2006). Body mass index, metabolic syndrome, and risk of type 2 diabetes or cardiovascular disease. J. Clin. Endocrinol. Metab..

[32] Azizi F (2010). Appropriate waist circumference cut-off points among Iranian adults: The first report of the Iranian National Committee of Obesity. Arch. Iranian Med..

[33] Zeinalian R, Farhangi MA, Shariat A, Saghafi-Asl M (2017). The effects of Spirulina Platensis on anthropometric indices, appetite, lipid profile and serum vascular endothelial growth factor (VEGF) in obese individuals: A randomized double blinded placebo controlled trial. BMC Complemen. Altern. Med..

[34] Sahaf R (2014). Validity and reliability of self-report physical activity Instruments for Iranian older people. Iran. J. Aging..

[35] Esfahani FH, Asghari G, Mirmiran P, Azizi F (2010). Reproducibility and relative validity of food group intake in a food frequency questionnaire developed for the Tehran Lipid and Glucose Study. J. Epidemiol..

[36] Asghari G (2012). Reliability, comparative validity and stability of dietary patterns derived from an FFQ in the Tehran lipid and glucose study. Br. J. Nutr..

[37] Kim J-O, Mueller CW (1978). Factor Analysis: Statistical Methods and Practical Issues.

[38] Xu X, Hall J, Byles J, Shi Z (2015). Dietary pattern is associated with obesity in older people in China: Data from China Health and Nutrition Survey (CHNS). Nutrients.

[39] Tinsley HE, Brown SD (2000). Handbook of Applied Multivariate Statistics and Mathematical Modeling.

[40] Czerkies M, Kwiatkowska K (2014). Toll-like receptors and their contribution to innate immunity: Focus on TLR4 activation by lipopolysaccharide. Adv. Cell Biol..

[41] Laugerette F, Vors C, Peretti N, Michalski M-C (2011). Complex links between dietary lipids, endogenous endotoxins and metabolic inflammation. Biochimie.

[42] Jialal I (2014). Rajamani UEndotoxemia of metabolic syndrome: A pivotal mediator of meta-inflammation. Metab. Syndr. Relat. Disord..

[43] López-Moreno J (2017). Effect of dietary lipids on endotoxemia influences postprandial inflammatory response. J. Agric. Food Chem..

[44] Villegas R, Salim A, Flynn A, Perry I (2004). Prudent diet and the risk of insulin resistance. Nutr. Metab. Cardiovasc. Dis..

[45] Eliassen AH (2013). Associations between red meat intake and biomarkers of inflammation and glucose metabolism in women. Am. J. Clin. Nutr..

[46] Martínez-González M (2015). Dietary inflammatory index and anthropometric measures of obesity in a population sample at high cardiovascular risk from the PREDIMED trial Running title: Dietary inflammatory index and obesity. Br. J. Nutr..

[47] Shivappa N (2015). Associations between the dietary inflammatory index and inflammatory markers in the Asklepios Study. Br. J. Nutr..

[48] Wirfält E (2001). Food patterns and components of the metabolic syndrome in men and women: A cross-sectional study within the Malmö Diet and Cancer cohort. Am. J. Epidemiol..

[49] Fung TT (2001). Association between dietary patterns and plasma biomarkers of obesity and cardiovascular disease risk. Am. J. Clin. Nutr..

[50] Williams DE (2000). A cross-sectional study of dietary patterns with glucose intolerance and other features of the metabolic syndrome. Br. J. Nutr..

[51] Maskarinec G, Novotny R, Tasaki K (2000). Dietary patterns are associated with body mass index in multiethnic women. J. Nutr..

[52] Schulz M (2000). Food groups as predictors for short-term weight changes in men and women of the EPIC-Potsdam cohort. J. Nutr..

[53] Slattery ML, Edwards SL, Boucher KM, Anderson K, Caan BJ (1999). Lifestyle and colon cancer: An assessment of factors associated with risk. Am. J. Epidemiol..

[54] Schulze MB, Fung TT, Manson JE, Willett WC, Hu FB (2006). Dietary patterns and changes in body weight in women. Obesity.

[55] Kim J, Jo I, Joung H (2012). A rice-based traditional dietary pattern is associated with obesity in Korean adults. J. Acad. Nutr. Diet..

[56] Mantzoros CS (1999). The role of leptin in human obesity and disease: A review of current evidence. Ann. Intern. Med..

[57] Zuo H (2014). Serum leptin concentrations in relation to dietary patterns in Chinese men and women. Public Health Nutr..

[58] Esmaillzadeh A (2007). Dietary patterns and markers of systemic inflammation among Iranian women. J. Nutr..

[59] Vahid F, Shivappa N, Hekmatdoost A, Hebert JR, Davoodi SH, Sadeghi M (2017). Association between Maternal Dietary Inflammatory Index (DII) and abortion in Iranian women and validation of DII with serum concentration of inflammatory factors: A case–control study. Appl. Physiol. Nutr. Metab..

[60] Wirth M (2014). Association of a dietary inflammatory index with inflammatory indices and the metabolic syndrome among police officers. J. Occup. Environ. Med..

[61] Hebert JR, Clemow L, Pbert L, Ockene IS, Ockene JK (1999). Social desirability bias in dietary self-report may compromise the validity of dietary intake measures. Int. J. Epidemiol..

[62] Hebert JR (1997). Gender differences in social desirability and social approval bias in dietary self-report. Am J. Epidemiol..

