# Supplementary material for: The association of dietary patterns with dietary inflammatory index, systemic inflammation, and insulin resistance, in apparently healthy individuals with obesity
Source: Sci Rep. 2021 Apr 6;11:7515. doi: 10.1038/s41598-021-86993-7 (PMC8024341; doi:10.1038/s41598-021-86993-7)
Supplement: Supplementary file 1 — Supplementary Information. [file 41598_2021_86993_MOESM1_ESM.docx]

**Title: Association of different dietary patterns with dietary inflammatory index, systemic inflammation, and insulin resistance in the apparently healthy obese**

Maryam Saghafi-Asl^1^, Susan Mirmajidi^2^, Mohammad Asghari Jafar-Abadi^3,4^, Farhad Vahid^5^, Nitin Shivappa^6,7,8^,James R. Hébert ^6,7,8^, Vahideh Ebrahimzadeh Attari^9^

^1^ Department of Clinical Nutrition, School of Nutrition and Food Sciences, Tabriz University of Medical Sciences, Tabriz, Iran.

^2^ Student Research Committee, Tabriz University of Medical Sciences, Tabriz, Iran.

^3^ Department of Statistics and Epidemiology, School of Health, Tabriz University of Medical Sciences, Tabriz, Iran.

^4^ Road Traffic Injury Research Center, Tabriz University of Medical Sciences, Tabriz, Iran.

^5^ Luxembourg Institute of Health, Luxembourg.

^6^Department of Epidemiology & Biostatistics, Arnold School of Public Health, University of South Carolina, Columbia, SC 29208.

^7^Cancer Prevention and Control Program, Arnold School of Public Health, University of South Carolina, Columbia, SC 29208.

^8^Connecting Health Innovations, LLC, Columbia, SC 29201.

^9^Department of Nutrition and Food Sciences, Maragheh University of Medical Sciences, Margheh, Iran

Correspondence to: **Susan Mirmajidi,**Student Research Committee, Tabriz University of Medical Sciences, Tabriz, Iran. Email: [susan_mirmajidi@yahoo.com](mailto:susan_mirmajidi@yahoo.com), Cellphone: +98-933-881-5138.

| **Appendix 1.** Classification of foods from FFQ based ontheir nutrient profile or culinary usage | |  |
| --- | --- | --- |
| **Food items** | | **Food groups** |
| White breads (lavash, baguette), dark breads (sangak, barbari, taftoon), noodles, pasta, rice, biscuit, barley, bulgur, cornflakes | | Grains |
| Beans, peas, lima beans, broad beans, lentils, soy | | Legumes |
| Potatoes | | Potatoes |
| French fries, potato chips | | French fries |
| Cabbage, cauliflower, Brussels sprouts, kale, cucumbers, mixed vegetable, eggplant, celery, green peas, green beans, green peppers, corn, turnips, squash, mushrooms, onions, garlic | | Vegetables |
| lettuce, spinach boiled | | Green leafy vegetables |
| Carrots row and boiled | | Yellow vegetable |
| Tomatoes, tomato sauce | | Tomatoes |
| Pears, apricots, cherries, apples, grapes, bananas, cantaloupe, watermelon, oranges, grapefruit, kiwi, strawberries, peaches, nectarines, tangerines, mulberries, plums, persimmons, pomegranates, lemons, pineapples, fresh figs, | | Fruits |
| Beef, hamburger, lamb, grained meat | | Red meat |
| beef liver, brain, heart, Tongue and organ meats | | Organ meats |
| Dried berries, raisins, dry fruits | | Dry fruits |
| sausages, | | Process meat |
| Chicken with or without skin | | Poultry |
| eggs | | Eggs |
| Canned tuna fish, other fish | | Fish |
| Skim or low fat milk, low fat yoghurt, dough | | Low-fat dairy products |
| High-fat milk, whole milk, chocolate milk, cream, high-fat yoghurt, cream yoghurt, cream cheese, other cheeses, ice cream, whey | | High-fat dairy products |
| Mayonnaise | | Mayonnaise |
| Hydrogenated fats, animal fats | | Hydrogenated fats |
| butters | | Butter |
| olives, olive oils | | Olive oil |
| Peanuts, almond, pistachios, hazelnuts, roasted seeds, walnuts | | Nuts |
| Sugars, candies, jam, jelly, honey, gaz, chocolate, halva, Chocolates, cookies, cakes, confections | | Sugars |
| Soft drinks | | Soft drinks |
| Tea, coffee | | Unsweatend drinks |
| apple juice, orange juice, grape fruit juice, other fruit juices, compote | | Fruit juice |
| Pickles, pickled cucumber, keraker | | Pickles |
| Pizza | | Pizza |
| FFQ: food frequency questionnaire | | |

| **Appendix2. Rotated Component Matrix^a^** | | | |
| --- | --- | --- | --- |
|  | Component | | |
|  | 1 | 2 | 3 |
| grains |  |  | .401 |
| legums |  | .115 | .493 |
| eggs | .243 | -.158 | .556 |
| fruit | .395 | .231 | .201 |
| nuts |  |  | .423 |
| sweets | .264 | .354 | .224 |
| hydrogenated.fat | .619 |  |  |
| fish | .156 |  | .432 |
| high.fat.dairy | .356 | .408 | .134 |
| low.fat.dairy |  |  | .703 |
| Unsweetened drink | .215 | -.192 | .261 |
| Procees meat |  | .338 | -.196 |
| pizza |  | .438 |  |
| French fries |  | .525 | .162 |
| potato | .205 | -.202 | .219 |
| olive |  |  | .200 |
| Greenleafy vegetable | .443 | .199 | -.205 |
| Yellow vegetable | .269 | .268 | .383 |
| tomato | .215 | -.142 | .219 |
| Othervegetable | .649 |  | .296 |
| poultry | .254 | .310 |  |
| Redmeat | .489 | .201 |  |
| Organ meat | .381 | .110 | .331 |
| Dryfruit |  | .104 | .242 |
| Soft drink |  | .761 | .184 |
| Butter |  | .485 | .392 |
| Mayonnise | .418 | .481 | -.231 |
| Fruit juic |  | .494 | .172 |
| Pickeles |  | .360 |  |
| Extraction Method: Principal Component Analysis.  Rotation Method: Varimax with Kaiser Normalization. | | | |
| a. Rotation converged in 9 iterations. | | | |


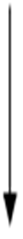


All participants entered into statistical analysis

n= 150

Missed

n= 20

N


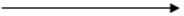


Missed because of insufficient data: 20


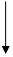


Excluded

n= 230


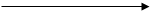


Enter to laboratory analysis

n= 170

not obese: 95

surgery during the past year: 25

corticosteroids users: 9

anti-obesity drugs users: 20

Recent use of anti-inflammatory drugs: 12

infectious disease: 8

hypertension: 43

Recent use of multivitamins: 5

Having kidney or liver diseases: 10

Not interested to participate: 3

Four-hundred volunteers were randomly invited from the general population.

**Supplementary Figure 1.**The flow chart diagram presenting the procedure of selecting sample population of obese subjects
